# National Physical Education

**Published:** 1920-03-06

**Authors:** 


### The Hospital

The Workers' Newspaper of Administrative Medicine and Institutional Life, Administration, National Insurance and Health.

No. 1761, Vol. LXVII. SATURDAY, MARCH 6, 1920. PRICE TWOPENCE

### NATIONAL PHYSICAL EDUCATION.

On another page (p. 533) we print an abstract of a paper read before the Royal Society of Medicine by Surgeon-Commander K. Digby Bell, R.N., the Medical Officer of the Royal Naval School of Physical and Recreational Training at Portsmouth, in which he outlined the scheme which is now in use at that school. The plan itself is the product of the brain of Commander B. T. Coote, R.N., the technical adviser of the school, and it is of so remarkable a nature and is likely to play such a profound part in the future of the British Empire and indeed in the advancement of mankind to the higher planes of civilisation, that we trust it will be widely read and meditated upon. We would further advise all who see and appreciate the true inwardness of what virtually amounts to the beginnings of a new social and moral movement, to visit the Naval School at Portsmouth for themselves, as we have done, and we will guarantee that any doubts which they may entertain as to the gigantic possibilities of the methods there employed will be dispelled.

The aims of physical education in the Navy are not confined merely to the production of a fine physique, great as such an object must be to a C 3 nation like ourselves, of whom it can be said that during a terrible period of national peril, but 36 per cent. of its manhood of fighting age was fit to go forth and meet the enemy in the gates. In addition to this aim, and intimately associated with it, a far higher object is pursued—namely, the development of strong and healthy character. This, of course, ought to be, though at present it is not, the effect of all education. Some partial efforts, it is true, have been made in this direction, notably by the boy scout movement, and to some extent also by the maintenance of those high traditions which render the Public Schools so much beloved. But for a great proportion of the masses no organised and efficient endeavour whatever is made to build up a sound foundation of good citizenship, integrity, and honour. Beyond such chance influences, which may be good, bad, or merely negative, as the individual's home affords, the average man's character at present is allowed to be moulded in the main by his primal instincts tempered by the proximity of the policeman.

Not a word can be said to justify this. Probably none would wish to make the attempt. And yet the inevitable consequence of the present lassitude, if allowed to continue, will be no less a thing than the lapse and decay of the British Empire. We shall meet the historic fate of all great nations. The collective spirit, the herd instinct, and its entailed willingness to self-sacrifice, will fade out from among us. The ever-growing desire of individuals to depend as parasites on the State, the dissolution of all bonds of discipline, these are the root evils which have brought about the downfall of the civilisations of the past. And we are bound to assume that our fate will be the same unless we have a care. What otherwise can be expected of a nation where a crowd of 60,000 people will gather to watch twenty-two men play unclean football, and will exercise their enjoyment of the sport mainly upon the odds of the betting? That is not recreation, nor does it help to mould fine character. It is the sign of a decadence that has already begun. What is the remedy?

Unhappily a lethargy has settled upon those in authority, and we look in vain for the strong arm of Government to take action. Last year the headmasters of our public schools included the subject of physical education in their annual conference. Subsequently, the president of the conference, in his capacity as such, approached the Board of Education to push for proper Government control with regard to experts of physical education; that is to say, the necessity for a National College of

Physical Education. He was, however, given clearly to understand that the Board of Education could not view matters in that light, and that such a development should be left to private enterprise. Furthermore, in May of last year the United Services held a conference on physical training, at which were present representatives of the Colonies and a member of the Board of Education. The proceedings ended in the formation of a sub-committee of which the member of the Board of Education was to act as chairman. This sub-committee was to be called as soon as information was received that the Board of Education had sanctioned their representative sitting as its chairman. Up to the present this sanction has been withheld. Manifestly the Board of Education are not prepared to give their help. It remains, therefore, to force the matter on the attention of the public. With this end in view we hope that every medical man in the country will interest himself in the matter and act as an apostle in a great cause.

